# Chemical-Functional Analysis of Extracts Obtained from *Zuccagnia punctata* Powder Using Green Solvents (NaDESs) in Conjunction with Traditional and Non-Traditional Techniques

**DOI:** 10.3390/plants13182563

**Published:** 2024-09-12

**Authors:** Antonela Mariana Romero, Iris Catiana Zampini, María Inés Isla

**Affiliations:** 1 Instituto de Bioprospección y Fisiología Vegetal (INBIOFIV), CONICET-Universidad Nacional de Tucumán (UNT), San Martin 1545, San Miguel de Tucumán T4000CWF, Argentina; anto_romero_@hotmail.com (A.M.R.); zampini@csnat.unt.edu.ar (I.C.Z.); 2 Facultad de Ciencias Naturales e IML, Universidad Nacional de Tucumán (UNT), Miguel Lillo 205, San Miguel de Tucumán T4000JFE, Argentina

**Keywords:** *Zuccagnia punctata*, green solvents, biocomposites, antioxidant activity, antifungal activity

## Abstract

*Zuccagnia punctata* Cav. (Family Fabaceae. Subfamily Caesalpinioideae) is a native plant species with a long history of use in Argentine traditional medicine. The purpose of the present study was to extract bioactive compounds with antioxidant and antifungal activity from *Z. punctata* aerial parts using conventional solvents (water, ethanol 60°, vegetal oil) and unconventional solvents (natural deep eutectic solvents or NaDESs) such as green solvents with and without the assistance of ultrasound (UAE) and microwaves (MAE). NaDESs such as glucose: lactic acid (LGH), sucrose: citric acid (CAS), choline chloride: urea (CU) and glucose: fructose: sucrose (FGS) were used. LGH and CU were effective in the extraction of phenolic compounds (6710 ± 10.12 µg GAE/mL and 7140 ± 15.00 µg GAE/mL, respectively) as well as ethanol (6270 µg ± 12.00 µg GAE/mL) using conventional methods. Two chemical markers of *Z. punctata* (2′,4′-dihydroxychalcone and 2′,4′-dihydroxy -3-methoxychalcone) were extracted in a high proportion in ethanol, oil, LGH and CU with UAE. The ABTS antioxidant capacity was higher in the extracts obtained with LGH and CU (SC_50_: 0.90 ± 0.10 µg GAE/mL and 1.08 ± 0.16 µg GAE/mL, respectively). The extract obtained with vegetal oil was the most potent as antifungal, followed by the extracts in ethanol, LGH and CU. These findings highlight the importance of using environmentally friendly solvents such as NaDESs to obtain bioactive metabolites from *Z. punctata,* an endemic plant of Argentina with a potential application in the food, cosmetic and pharmaceutical industries.

## 1. Introduction

*Zuccagnia punctata* Cav., commonly known as jarilla pispito, pus pus, and jarilla macho, is a medicinal shrub that grows in Argentina’s northern Monte desert, a fragile ecosystem, highly vulnerable to degradation [1,2,3,4,5]. Aerial parts of this shrub are used in traditional medicine by different communities as infusions, decoctions in water and macerations in ethanol to treat fungal and bacterial infections, asthma, arthritis, inflammations, and tumours [6]. Antibacterial activity against antibiotic-resistant strains was demonstrated for *Z. punctata* aqueous and ethanolic extracts [7,8,9,10]. An effect on yeast such as *Candida albicans* and *Cryptocccus neoforms* was also demonstrated [11,12,13,14]. Butassi et al., 2015 [15] described the synergistic effect between *Z. punctata* dichloromethane (DCM) extract and *Larrea nitida* DCM extract, another plant species that grows in Argentine northern deserts, on *C. albicans* and *C. glabrata* growth. Nuño et al., 2014 [12] showed that *Z. punctata* DCM extracts are effective as inhibitors not only for *Candida* growth and biofilm formation but also for preformed *Candida* biofilm and yeast germ tube formation, in doses lower than the minimal inhibitory concentration (MIC). *Z. punctata* chloroformic and methanolic extracts were also active on dermatophyte fungi isolated from skin infections (*Microsporum gypseum*, *Tricophyton rubrum* and *Tricophyton mentagrophytes*) [11]. The main bioactive compounds identified in *Z. punctata* extracts were 2′,4′-dihydroxy-3′-methoxychalcone and 2′,4′-dihydroxychalcone [16].

Up to now, *Z. punctata* extracts used to demonstrate antimicrobial activity have been obtained by conventional extraction methods (CEM) i.e., maceration or infusion or using organic solvents [11,12,13,15,16]. However, these processes require long extraction times, high temperatures, high amounts of solvent, and energy consumption to remove the solvent by evaporation or lyophilization and some of the solvents used are harmful to human health and the environment [17]. A growing awareness of the human impact on the environment has promoted ‘green extraction’ as an alternative process to organic extraction. Consequently, an area of research in the development of green extraction is dedicated to the design of new environmentally friendly solvents that meet both technological and economic demands [18].

The compounds, mainly sugars, organic acids, choline derivatives, or urea, when combined in a certain proportion, form a new deep eutectic liquid phase referred to as Natural Deep Eutectic Solvents (NaDESs) [19]. One of the most important advantages of NaDESs is the possibility of preparing these solvents easily, apart from the large number of combinations that could be applied. These solvents are formulated by combining a hydrogen acceptor, typically choline chloride (ChCl), and a hydrogen bond donor such as carboxylic acids, sugars, amino acids, or other naturally occurring compounds [20,21]. Both acceptor and donor are generally in a solid state and, under the right conditions, give rise to a liquid, even at low temperatures, with a lower melting point than each of the other compounds separately (eutectic mixture) [20,22,23,24,25]. The striking trait of these solvents is their ability to donate and accept protons and electrons by forming hydrogen bonds, thus increasing their capacity for both extraction and dissolution [26]. One of the advantages for the application of NADESs as a green extraction solvent is that the properties described above can be easily adapted by either changing the selection of components or the molar ratio between the components, and by adding water [22]. This homogenous liquid is stable once the eutectic point is reached, making it easy to use for bioactive compound extraction from plant tissues. NaDESs are stable and can extract both hydrophilic and hydrophobic compounds from the plant matrix [22,27]. In particular, the extraction efficiency of phenols, terpenoids and other compounds increased when NaDESs were employed instead of traditional solvents. Several studies have reported the successful application of NaDESs in the extraction of phenolic compounds, indicating their great potential in the production of plant extracts for direct use in human consumption [22,27,28,29,30]. These properties open a gate to developing innovative extracts with unique phytochemical profiles and biological activities as regards pharmaceutical and/or cosmetic products.

On the other hand, in addition to the characteristics of the solvent to be used, another significant factor to consider in order to obtain more molecule extraction from complex matrices is the method used. Ultrasound-assisted extraction (UAE) and microwave-assisted extraction (MAE) are non-conventional extraction methods (NCEM) that have several advantages, such as shorter extraction times, smaller solvent volume used, and higher extraction yields and are considered environmentally friendly technologies [31,32].

**Figure 1:**
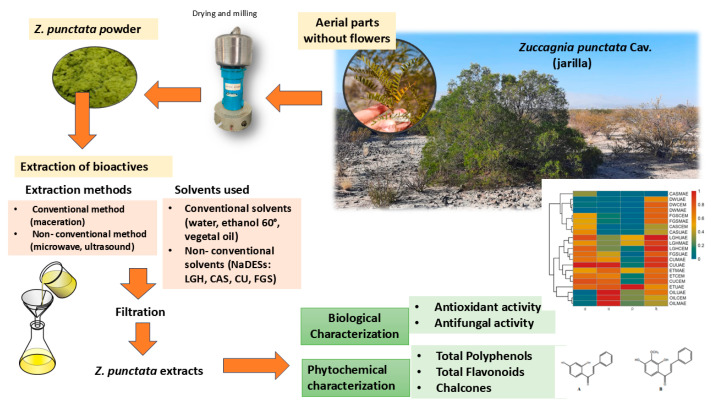
Flowchart of procedure to obtain *Z. punctata* extracts using water, ethanol, vegetal oil and NaDESs as lactic acid: glucose (LGH), sucrose: citric acid (CAS), choline chloride: urea (CU) and fructose: glucose: sucrose (FGS), assisted by microwave and ultrasound. Antioxidant and antifungal activity characterization.

Therefore, the aim of the present study was to extract phenolic compounds (PC), principally chalcones, from *Z. punctata* aerial parts using different NaDESs as solvents, with and without UAE and MAE, and to compare them with extractions using CEM and solvents such as vegetal oil, ethanol and water (Figure 1). The extraction efficiency of chalcones was determined, and the antioxidant and antifungal activity against dermatophyte fungal strains was also analyzed. To the best of our knowledge, no specific studies on the advantage of NaDESs over conventional solvents regarding the extraction of phenolic compounds from aerial parts of *Z. punctata* have been conducted so far.

## 2. Results and Discussion

In general, the extraction capacity for different classes of PC from plant material can potentially be influenced by both the extraction conditions (methodology, temperature, plant material/solvent ratio) and by the physicochemical properties of the solvent used. For this, the conditions cannot be generalized for all plant materials due to the diverse nature of the bioactive compounds. Some methods were previously reported for phenolic compound extraction (PCE) from *Z. punctata* aerial parts, i.e., maceration with organic solvents and water [6,7,8,9,10,11,12,13,14,15,33,34]; however, the use of non-conventional solvents such as NaDESs have not been described up to the present moment. Furthermore, the use of NCEMs such as UAE or MAE to extract bioactive compounds from aerial parts of *Z. punctata* have not been described either. Therefore, this work focuses on the use of four NaDESs. The combination of deep eutectic solvents based on organic acids (LGH, CAS) or choline chloride (CU) and sugars (FGS) was chosen to study its behavior to obtain PC-enriched extracts from the powder of *Z. punctata*. The selected NaDESs have different pHs and viscosities. The ratio in the preparation of the individual NaDES mixtures is given in the Section 3. The efficiency of the PC and chalcone extraction using NaDESs was compared to other solvents, i.e., distilled water (DW), ethanol and vegetal oil. Hence, *Zp* extracts were obtained from aerial parts of *Z. punctata* as the flux diagram in Figure 1 depicts.

### 2.1. Yield of Phenolic and Flavonoid Compound Extraction Using Conventional and Non-Conventional Solvents, with and without UAE or MAE

According to our experimental conditions and using validated spectroscopic methods as described in the Section 3, the yield of PC obtained by extraction of *Z. punctata* powder without ultrasound and microwave ranged from 1624 to 7140 µg GAE/mL of extract (yield of 0.16 to 0.71%) (Table 1). It is evident that CU consisting of choline chloride and urea as a hydrogen bond donor was not only the best among the tested NaDESs (0.84%) followed by LGH, but also the green solvent with similar extraction capacity with respect to ethanol and the highest of vegetal oil and water. Several works have reported the highest efficiency in the extraction of PC from different plant materials using NaDESs based on choline chloride, with respect to other mixtures of NaDESs and organic solvents [35,36,37,38,39,40]. The high performance of these NaDESs is due to the strong molecular interaction between choline chloride and PC with the availability of a hydrogen bond formation, which leads to a multiple interaction network [39]. Mansinhos et al., 2021 [41] reported that PC extraction from *Lavandula pedunculata* that was CU-assisted using ultrasound was more efficient than maceration without ultrasound.

CAS and FGS were the NaDESs with the lowest extraction potency of phenolic compounds. This could be due to the high viscosity of these solvents (140 and 720 Pa.s, respectively). Other authors have shown that a factor of great influence on the extraction yield is the viscosity of these solvents, a fact which makes it difficult to transfer mass from the solid matrix to the solution [26,40].

In our study, the ultrasound and microwaves do not improve, in general, the efficiency of extraction of *Z. punctata* polyphenols. Martinez Chamás et al., 2023 [42] reported similar results for PC extraction from *Fabiana* sp. using maceration and UAE.

The solvents that showed the lowest extraction efficiency of PC from *Z. punctata* were water and oil.

The order of flavonoid extraction performance using MAE and UAE with different solvents was the following: oil > CU > ethanol > LGH > FGS > CAS = DW. CU showed a higher extraction capacity of flavonoids than ethanol 60° and lower than oil as a solvent (Table 1). In our study, the UAE using oil or CU as solvent was the most promising extraction method for flavonoids. The solvents that showed the lowest flavonoid extraction efficiency were DW and CAS. No significant differences were found in the content of phenolic compounds and flavonoids using plant material collected in the same place and month but different years (between 2021 and 2023) using the same conditions of extraction. The results of TPC and FC obtained with the material collected in each year are shown in the Supplementary Materials section (Tables S1–S3). Table 1 shows the average TPC and FC per mL of each extract obtained using the same solvent and methodology with plant material obtained over the three years. These results allow us to demonstrate the chemical stability of these compounds in the plant material, which is important when selecting storage conditions for the conservation of the plant material to be used for extractions of bioactive compounds. Therefore, this is the first extraction study of phenolic compounds from *Z. punctata* that has focused on the use of green solvents and MAE and UAE compared to traditional methods.

### 2.2. Yield Chalcones Extracted from Z. punctata with Green Solvents and Other

The 2′,4′-dihydroxy-3′-methoxychalcone (DHMC) and 2′,4′-dihydroxychalcone (DHC) are the main constituents of aqueous and ethanolic, methanolic and DCM extracts and could be considered as markers for the chemical compounds of extracts from aerial parts of *Z. punctata* [6,7,12,13,33,43]. The DHC showed antibacterial capacity with MIC values between 0.10 and 100 µg/mL [7] and between 50 and 500 µg/mL against different Gram-positive and Gram-negative bacteria, respectively [9,33]. The DHC also inhibited biofilm formation [33]. Antifungal and anti-inflammatory activities were also attributed to isolated DHC from *Z. punctata* [11,12,13,14].

This is the first report on DHC and DHMC extraction using NaDESs with and without microwave and ultrasound assistance. For the quantification of chalcones, a validated HPLC-DAD method was used as described in materials and methods.

**Figure 2:**
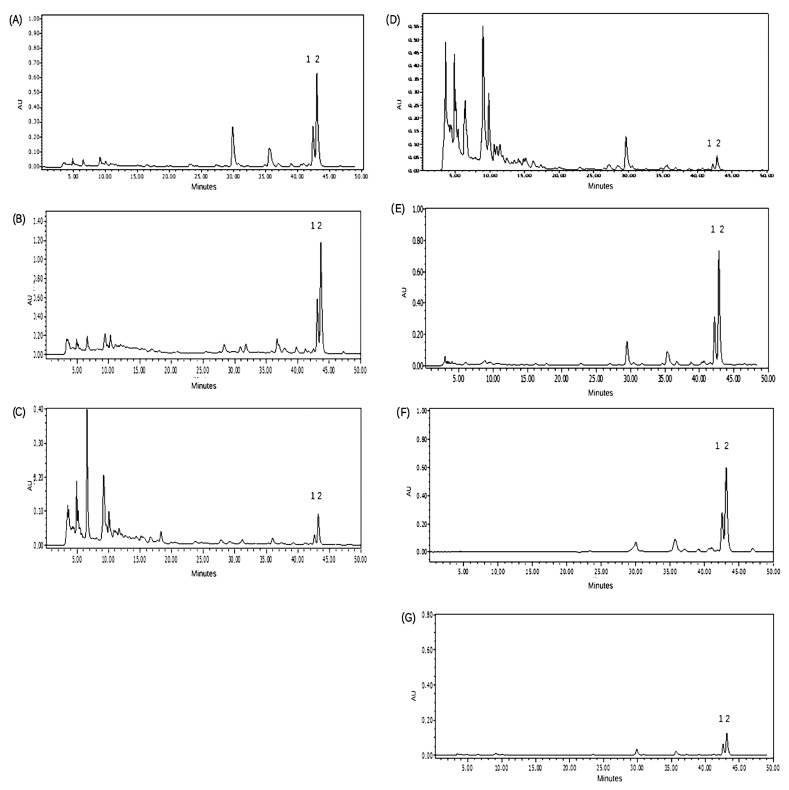
HPLC-DAD profiles of conventional extraction method (CEM) without microwave-assisted extraction (MAE) or ultrasound-assisted extraction (UAE) using different solvents acquired at 330 nm. (**A**) LGH (dilution 1/2), (**B**) CU, (**C**) FGS, (**D**) distilled water, (**E**) Ethanol 60° (dilution 1/5), (**F**) Vegetal oil (dilution 1/7), (**G**) CAS. Peak 1: 2′, 4′-dihydroxy chalcone (DHC); Peak 2: 2′,4′-dihydroxy-3′-methoxychalcone (DHMC).

The HPLC-DAD profiles were analyzed for all the obtained extracts using different extraction methods and different solvents (Table 2, Figure 2 and Figure S1). Similar HPLC-DAD profiles were observed for extracts in CU and LGH, both NaDESs, as well as in ethanol and in the oil extract (Figure 2A,B,E,F). The best solvents to extract chalcones without ultrasound and microwave assistance were ethanol, followed by vegetal oil and the two NaDESs, LGH and CU (Table 2). The extraction capacity of DHC and DHMC with ethanol improved with the assistance of ultrasound by around 1.64 times. Choommongkol et al., (2022) [44] found that MAE improves the extraction of 2′,4′-dihydroxy-6′-methoxy-3′,5′-dimethyl chalcone from *Syzygium nervosum* fruits, obtaining higher yields than with extraction by maceration with methanol or conventional heat reflux. In *Z. punctata* extracts obtained with LGH as the solvent, however, the chalcone yield improves four times with the assistance of ultrasound and microwaves probably because of their low viscosity and better mass transfer during the extraction. The heating of LGH using MAE at 50 °C and UAE at 60 °C probably also promotes a significant increase in the recovery of chalcones in comparison with ethanol. A similar result was obtained by Obluchinskaya et al. 2021 [45] with several hydrophilic and lipophilic compounds extracted from *F. vesiculosus* with NaDES using UAE.

The extraction performance of chalcones with CU using CEM is like that obtained with LGH, but the performance does not improve with the assistance of ultrasound or microwaves. Other authors reported the same behavior for CU in extraction of PC from sour cherry pomace [46]. A poor extractability of prenylated chalcones (isosalipurposide and tomoroside A) from *Helichrysum arenarium* using NADESs containing choline chloride as the solvent was demonstrated when compared to maceration in MeOH 80% [47].

The extraction of chalcones from *Z. punctata* powder with FGS and CAS was lower than the other NaDESs and did not improve when using microwave or ultrasound probably due to its high viscosity (720 and 140 Pa.s, respectively). Moreover, the decrease in viscosity could be beneficial to improve the cavitation effect of the ultrasonic waves and reduce the loss of ultrasonic waves in the process of propagation. The lowest chalcone extraction yield was obtained with distilled water and FGS. The HPLC-DAD profiles for the aqueous extracts and the extracts in FGS showed a greater number of peaks corresponding to more hydrophilic components than chalcones (Figure 2C,D).

### 2.3. Antioxidant Activity

Previously, the antioxidant capacity on radical cation ABTS for infusion or tea (aqueous extract) made with *Z. punctata* aerial parts was previously demonstrated [34]. These infusions also showed hydroxyl and O• radicals and H_2_O_2_ scavenging capacity and lipid peroxidation protection [6]. However, the extraction performance of the active ingredients in the infusion is poor. Several authors also reported that *Z. punctata* extracts and PC isolated from them with organic solvents are able to scavenge free radicals [13,43,48,49]. However, organic solvents pollute the environment, leaving behind toxic residues. For this reason, it is necessary to look for alternative environmentally friendly solvents and methods for the efficient extraction of plant antioxidants that can be used in cosmetics, food, pharmacy and even agri-business such as NaDESs. In the present work, the antioxidant activity of *Z. punctata* extracts obtained with NaDESs and conventional solvents such as water, oil and ethanol were compared for the first time. The biological activity was also determined for the extraction solvents. None of the solvent controls showed antioxidant activity. The highest antioxidant activity on ABTS^•+^ was shown for extracts obtained with the LGH followed by extracts obtained in CU (Table 3). The ethanolic extract and DW extracts were the most active, with a potency similar to CU (SC_50_ = 1.5; 1.45 and 1.43 µg GAE/mL, respectively).

### 2.4. Correlation between Total Phenolic Compounds, Flavonoids, Chalcone Content and Antioxidant Activity

The Pearson correlation coefficient was used to evaluate the relationship between the antioxidant capacity (ABTS) and the content of total phenolic compounds (TPC), total flavonoids (TF) and the content of chalcones (CL) of the *Z. punctata* extracts. The TF showed a positive correlation in ABTS antioxidant activity (r = 0.96). Furthermore, the ABTS assay shows a significant correlation in TPC and CL content with R values of 0.43 and 0.63, respectively. Chalcone content shows a positive correlation with TF (r = 0.47).

A heat map with a dendrogram was made. This study uses a data visualization technique that measures the magnitude of a phenomenon of colors in two dimensions. Color variation can be expressed by tone or intensity, where similar rows and columns are grouped together.

**Figure 3:**
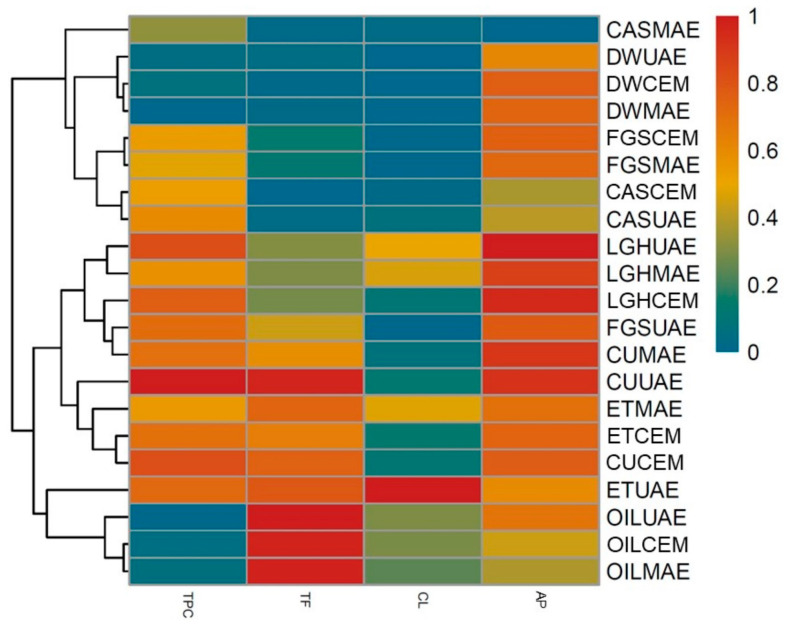
Heatmap and dendrogram of the total phenolic content (TPC), total flavonoid (TF), total chalcones (CL), and antioxidant power (AP) of extracts obtained with conventional and non-conventional method. MAE: microwave-assisted extraction; UAE: ultrasound-assisted extraction; CEM: conventional extraction; DW: distilled water; ET: ethanol; NaDESs: LGH (lactic acid: glucose), CAS (sucrose: citric acid), CU (choline chloride: urea) and FGS (fructose: glucose: sucrose).

It is separated into three groups, the first one being made up of CAS-MAE, DW-UAE, DW-CEM, DW-MAE, FGS-CEM, FGS-MAE, CAS-CEM, CAS-UAE, the second made up of LGH-UAE, LGH-MAE, LGH-CEM, FGS-UAE, CU-MAE, CU-UAE, ET-MAE, ET-CEM, CU-CEM and the third group made up of ET-UAE, OIL-UAE, OIL-CEM and OIL-MAE (Figure 3).

The first group is characterized by its low concentration of total flavonoid compounds and total chalcones, and an average antioxidant capacity. The second group presents a high concentration of total phenolic compounds, as well as flavonoids, a low concentration of total chalcones and a high antioxidant capacity. The third group features a low concentration of phenolic compounds, a very high concentration of flavonoid compounds, a medium/high content of total chalcones, as well as an average antioxidant capacity.

Group 2 showed the best performance as antioxidants. The extraction method does not have an influence on the differentiation of groups, a fact that was not observed when varying the extraction solvent.

### 2.5. Antifungal Activity

**Figure 4:**
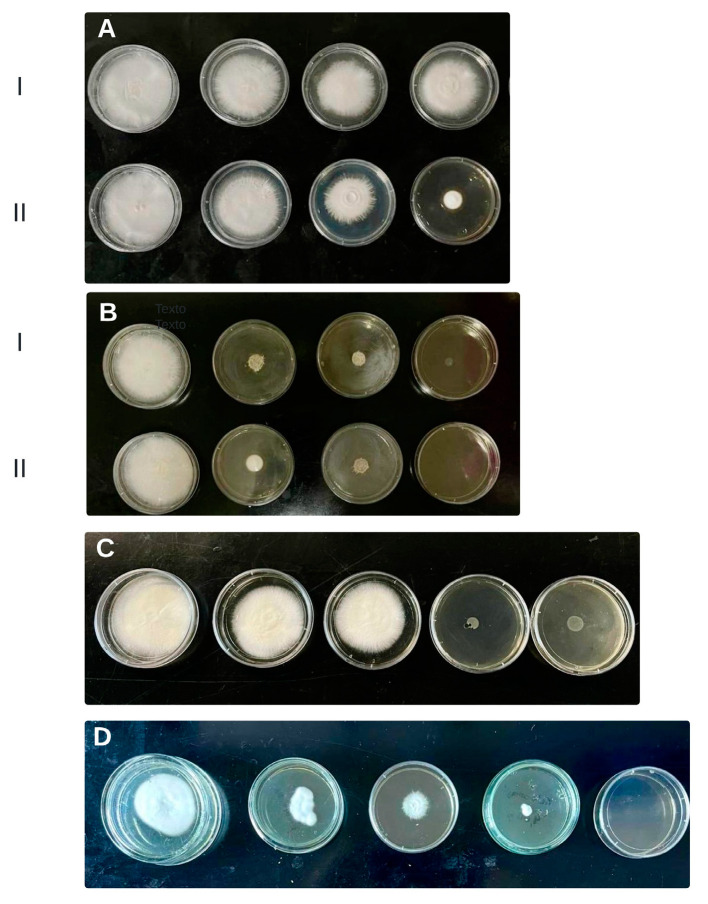
Agar macrodilution method of *Z. punctata* extracts against *T. mentagrophytes*. (**A**) I: CU Solvent control; II: Extract obtained with CU: strain control, 31.25 µg GAE/mL, 62.50 µg GAE/mL, 125 µg GAE/mL (CIM 125 µg GAE/mL). (**B**) I: CAS Solvent control II: Extract obtained with CAS: inactive (The solvent CAS was active); (**C**) 2′, 4′-dihydroxychalcone: strain control, 1.6 µg DHC/mL; 3.2 µg DHC/mL, 6.4 µg DHC/mL; 12.8 µg DHC/mL (CIM 6.4 µg DHC/mL). (**D**) ketoconazole: strain control, 1 µg/mL, 2 µg/mL, 4 µg/mL, 8 µg/mL (CIM 8 µg/mL).

Cutaneous fungal infections are mainly caused by keratinophilic filamentous fungi (dermatophytes), that use keratin as a nutrient in skin, hair and nail infections [50]. Dermatophyte species such as *Trichophyton* account for as many as 70% of dermatophytosis worldwide [51]. Although conventional antifungals are available, including azoles, allylamines, and morpholine derivatives, several side effects contribute to therapy failure [51]. For this, natural products could represent an alternative therapy for dermatophytosis [50,51,52]. Previously, the effect of *Z. punctata* methanolic and chloroform extracts on yeast such as *C. albicans* and dermatophyte fungi isolated from skin infections (*Microsporum gypseum*, *T. rubrum* and *T. mentagrophytes*) was demonstrated [11,12,13]. In this work, the activity of each extract of *Z. punctata* obtained with green solvents was tested in comparison with its corresponding solvent controls on *T. mentagrophytes* (Figure 4). The CAS NaDES showed an inhibitory effect on fungal growth due to the osmotic effect of sucrose. Other authors had previously reported the same behavior [53,54]. However, the compounds extracted with CAS were not active on fungal growth.

The extracts of *Z. punctata* obtained using the conventional method with oil, ethanol, LGH and CU as the solvent showed a high inhibitory capacity on the growth of *T. mentagrophytes* with the lowest MIC values (Table 4). As explained previously, these extracts are enriched with chalcones and total flavonoids. The MIC values of the ethanolic extract obtained by CEM and CEM assisted by microwave and ultrasound decreases (62.50; 31.17; 15 µgGAE/mL, respectively) probably due to an increase in the amount of extracted bioactive chalcone (Table 2). Previous work indicates that ethanolic extracts contain other chalcones in addition to DHC and DHMC, as well as flavones and flavanones and derivatives of caffeic acid [13,16]. The extracts obtained with FGS and DW as solvents did not show an antifungal effect, probably due to the low level of chalcones and total flavonoids. It is notable that the MIC value obtained for chalcone isolated from *Z. punctata* (6.4 µg/mL) was similar to the MIC value of the commercial antifungal, ketoconazol.

## 3. Materials and Methods

### 3.1. NaDESs Preparation

The NaDESs were prepared by mixing the components in the appropriate mole ratios, as follows: CAS, LGH, FGS and CU were prepared according to Dai et al. (2013a) and Kamradt Savi et al. (2018) [22,40] (Table 5). CAS and LGH were soaked in an ultrasonic bath at 40 °C for 120 min. FGS and CU were taken to the magnetic stirrer for 20 min and 1 h, respectively. The procedure was conducted at 40 °C and 80 °C to FGS and CU, respectively. The reagents were acquired in Cicarelli, Santa Fe, Argentina (ethanol, sucrose), Anedra, Bs As, Argentina (glucose, citric acid, urea), Sigma-Aldrich, Beijing, China (choline chloride 98.9% purity and DL-lactic acid 91.4% purity).

### 3.2. Plant Material

Aerial parts of *Z. punctata* were collected in February 2021, 2022 and 2023 from Argentinean Northwest in Ampimpa (26°35′33.3″ S 65°51′25.0″ W; 2329 masl), Amaicha del Valle, Tucumán. Voucher specimens were deposited at the Herbarium of Fundación Miguel Lillo, Tucumán, Argentina (LIL; Thiers, 2022): Ampimpa (LIL 618078). The identification of plant materials was carried out by the botanist Dr. Ana Soledad Cuello. The samples were dried until constant weight in a forced-air oven at 40 °C and then ground in a Helix mill (Numak, F100 Power 1/2 HP-0.75 Kw, Brusque, Brazil). The powder was kept in vacuum-sealed bags at 24 °C.

### 3.3. Conventional Extractions

Different extractive solutions were prepared (Table 6). Ground plant material (1 g) of different years between 2021 and 2023 was extracted in 20 mL ethanol, oil or NaDESs at 40 °C for 30 min in an ultrasonic bath (Ultrasonic Washer Arcano Model PS-10A, Shandong Arcano Ultrasonic Technology Co., Ltd., Jinan, China). The aqueous extracts were prepared with 1 g powder in 20 mL water at 100 °C for 10 min. Then, the extracts were vacuum filtered. The filtrate fraction of the aqueous extracts was frozen at −80 °C and were subsequently freeze dried (freeze dryer, RIFICOR, model L-M10-A-E50-CRT, Buenos Aires, Argentina). The ethanolic extracts were dried in a rotatory evaporator and freeze dried. The dried aqueous and ethanolic extracts were kept at −20 °C until use. The dried extracts were resuspended in ethanol and water at the time of use. The preparations in oil and NaDESs were kept at −20 °C until use.

### 3.4. Non-Conventional Extractions

#### 3.4.1. Ultrasound Probe-assisted Extraction (UAE)

The extraction mixtures, 1 g ground plant material of different years between 2021 and 2023 with 20 mL solvent (water, ethanol, oil and NaDESs) were placed in the ultrasonic probe (UP200St 200 W, 26 kHz, Hielscher Ultrasonics. GmbH., Teltow, Germany) and extracted in 3 cycles of 16 s on and 1 min off each one at a temperature between 24 °C and 55 °C, with an amplitude of 40% and a maximum power of 60 W (Table 6). Then, it was vacuum-filtered. The extractions were carried out in triplicate.

#### 3.4.2. Microwave-Assisted Extraction (MAE)

The extraction mixtures, 1 g of ground plant material of different years between 2021 and 2023 with 20 mL of each solvent were placed in contact in a digital microwave (BGH B120db920I, Buenos Aires, Argentina) in 3 cycles of 16 s on and 1 min off at a temperature of 60 °C and a power of 60%, and subsequently vacuum filtered (Table 6). The extractions were carried out in triplicate.

### 3.5. Determination of Chemical Composition

The quantification methods of phenolic, flavonoid, and chalcone compounds were validated as per the ICH guidelines [55] and are similar to the methods reported by laboratory on UV–Visible spectrophotometer method development and validation for herbal drugs [56,57]. The analyzed parameters for validation of methods were specificity, linearity, average recovery or accuracy; limits of detection and quantification (LOD and LOQ); and precision (repeatability). The reference compounds and analyzed parameters are included in Table 7.

#### 3.5.1. Total Polyphenol and Flavonoid Content Determination

The total phenolic compound content was determined using Folin–Ciocalteau reagent [58]. The blue color developed was read at 765 nm in UV/visible spectrophotometer (Jasco v-630, Thermo Fisher Scientific, Tokyo, Japan). Total flavonoids were estimated using the Woisky and Salatino method with AlCl_3_ reagent [59]. The solvent controls (DW, ethanol and NaDESs) were performed in each determination to scan any possible interference. The determinations were performed in triplicate in all extracts and solvents and the results were expressed as μg of gallic acid equivalent (GAE) per milliliter (μg GAE/mL) and quercetin equivalents (QE) per milliliter (μg QE/mL) for polyphenols and flavonoids, respectively. Folin–Ciocalteu reagent, AlCl_3_ and reference compounds (gallic acid, and quercetin) were acquired at Sigma Aldrich, St. Louis, MO, USA. The extraction yield in each solvent was determined as the amounts of chemical components per mL of extract.

#### 3.5.2. HPLC-DAD

All extracts were used in different dilutions according to phenolic compound content. The phenolic compounds of *Z. punctata* extracted with vegetable oil were recovered with ethanol and used to quantify the chalcones. The chromatographic method to determine Chalcones was validated, and the analyzed parameters were included in Table 7. The HLPC-DAD system used was a Waters 1525 equipment with a Waters 1525 binary pump system, a manual injection valve (Rheodyne Inc., Cotati, CA, USA) with 20 µL loop, a column thermostat compartment, and a Waters 2998 diode array detector. The analysis was performed at a temperature of 40 °C, using a 155 × 4.6 mm XBridge™ C18 (5 µm) column with a flow rate of 0.8 mL/min (Waters Corporation, Milford, MA, USA). The solvent system used for the separation of components from extracts was composed of solvent A (0.1% acetic acid in water) and solvent B (0.1% acetic acid in methanol) (conditions: 10–57% B from 0 to 45 min and kept at 100% B from 45 to 60 min). The flow rate was set at 0.5 mL/min. Data collection was carried out with Empower ^TM^ 2 software. The identification of phenolic compounds was carried out by comparing the retention times and spectral data (220–600 nm) of each peak with those of standards, 2′,4′-dihydroxy-chalcone (DHC) and 2′,4′-dihydroxy-3-methoxychalcone (DHMC) from Indofine SRL. The quantification of both chalcones was based on external calibration curves from available standards. Plots were built by comparison of area and concentration in the range of 1–500 ppm.

### 3.6. Antioxidant Activity Determination

#### 3.6.1. ABTS Free Radical Scavenging Activity

The antioxidant capacity assay was carried out by the improved ABTS^•+^ spectrophotometric method according to Re et al. (1999) [60]. In this method, ABTS^•+^ solution (Sigma Aldrich, St. Louis, MO, USA) was mixed with different amounts of the extractive solution obtained from *Z. punctata* powder. Solvent controls (water, ethanol, NaDESs) were performed in each determination to detect some possible interferences. Absorbance was recorded at 734 nm after 6 min. Results are expressed as SC_50_ values (μg GAE/mL), defined as the concentration of phenolic compounds necessary to scavenge the 50% of ABTS free radicals. Quercetin was used as a reference compound.

#### 3.6.2. Antifungal Activity Determination

##### Microorganism and Medium

The fungal strain, *Trichophyton mentagrophytes* (Tm_15_) (n = 1) was provided by Hospital del Niño Jesús, Tucumán, Argentina and identified by hospital staff by molecular phylogenetic techniques, and physiological and morphological analysis. The strain was included in institutional collection as INBIOFIV-Tm_15_. The fungus was grown in a solid medium Sabouraud agar with chloramphenicol (SA-Ch). The medium compositions were as follow: Sabouraud agar: meat peptone 1%, glucose 4%, agar–agar 1.5%, chloramphenicol 0.005%, pH 5.4–5.6.

##### Inocula

The inocula of Tm_15_ was prepared from a culture of 7 to 15 days of growth on Sabouraud Agar at 28 °C. For the collection of the conidia, the colony was scraped with a Pasteur pipette and to facilitate the dispersion sterile physiological solution containing Tween 20 (0.05%) was added. The suspension was adjusted to 0.5 of the McFarland scale and counted in Neubauer chamber (2.5 × 10^4^ CFU/mL) [61,62].

##### Minimal Inhibitory Concentration Determination

The evaluation of the antifungal potency of the extracts was carried out by means of the determination of minimum inhibitory concentration (MIC) values by macrodilution in solid medium [61]. The determination of the MICs was carried out in a volume of 5 mL of Sabouraud agar medium. Serial dilutions of all extracts in each solvent, i.e., distilled water, ethanol and NaDESs were prepared in a concentration range of 500 to 1.87 µg GAE/mL. The inoculum with a final concentration of 2.5 × 10^4^ CFU/mL was sown on the plates. Sterility controls of the culture medium (without inoculum), fungal growth control (without extract) and controls of each solvent (inoculum plus solvent) were carried out. The pH effect of solvent on microbial growth was also determined adjusting the culture medium pH between 2 and 6. Different commercial sugar solutions such as fructose, glucose and sucrose were diluted and added to the culture medium to obtain the same sugar concentration as NaDESs. In addition, ketoconazole (8 to 64 µg/mL) was used as a positive control. Then, the plates were incubated at 30 °C for 7 days.

### 3.7. Statistical Analysis

For the statistical analysis of the data, the Tukey test was applied, with a level of significance *p* > 0.05, using the statistical package InfoStat V1.1 [63]. The heatmap with dendrogram using Euclidean distance and Ward’s clustering algorithm was applied to the standardized dataset by using R software 3.0.2 (R studio Team, 2020 [64]).

## 4. Conclusions

We propose a new environmentally friendly methodology using NaDESs as the solvent for the extraction of phenolic compounds and chalcones from the aerial parts of *Z. punctata*. The extract obtained from the aerial parts presents an activity similar to or greater than the ethanolic or vegetable oil extract. LGH- and CU-based NaDESs were shown to be excellent overall extraction media and were especially suitable for the extraction of total phenolic compounds and flavonoids of *Z. punctata* with antioxidant and antifungal activity. Extraction with NaDESs has already been combined with two procedures: UAE and MAE, to improve extraction yield. These findings highlight the importance of using green solvents such as NaDESs to obtain bioactive compounds from plants with prospective applications in the cosmetic and pharmaceutical industry. Developing antifungal pharmaceutical formulations incorporating the *Zuccagnia punctata* extracts obtained with NaDESs in gels, creams or aerosols is to be expected in the near future. This could be an alternative for the treatment of pathologies related to fungi that cause mycosis in nails and skin.

## Figures and Tables

**Table 1:** Content of total phenolic compounds (TPC) and total flavonoids (TF) *in Z. punctata* extracts obtained with different solvents and conventional and non-conventional extraction methods from plant material collected between 2021 and 2023. Table 1 was made with respect to the data reported in the Supplementary Material (Tables S1–S3).

Solvents	CEM	UAE	MAE	CEM	UAE	MAE
	TPC (µg GAE/mL)			TF (µg QE/mL)		
DW	1762 ± 17.21 ^aB^	1582 ± 14.60 ^aB^	1258 ± 11.00 ^aA^	65.80 ± 15.00 ^aA^	85.10 ± 6.00 ^aA^	69.18 ± 2.00 ^aA^
E-60°	6270 ± 12.00 ^cB^	6470 ± 19.80 ^cB^	5160 ± 15.08 ^cA^	551.80 ± 16.09 ^dA^	660.50 ± 6.80 ^dA^	624.00 ± 5.70 ^eA^
Vegetal oil	1624 ± 18.30 ^aA^	1331 ± 12.30 ^aA^	1716 ± 7.50 ^aA^	806.70 ± 12.45 ^fA^	824.70 ± 8.00 ^eA^	809.40 ± 3.89 ^fA^
LGH	6710 ± 10.12 ^cB^	6350 ± 15.00 ^cB^	5380 ± 11.30 ^cA^	271.00 ± 18.00 ^cA^	289.5 ± 6.00 ^bA^	280.00 ± 2.50 ^cA^
CAS	5050 ± 20.80 ^bB^	5580 ± 13.00 ^bB^	3600 ± 12.03 ^bA^	52.70 ± 5.80 ^aA^	82.80 ± 6.03 ^aA^	67.50 ± 0.70 ^aA^
CU	7140 ± 15.00 ^dB^	8390 ± 27.80 ^dC^	6250 ± 12.00 ^dA^	637.00 ± 18.83 ^eA^	802.50 ± 8.03 ^eB^	508.50 ± 4.14 ^dA^
FGS	5070 ± 12.70 ^bB^	6340 ± 20.00 ^cC^	4250 ± 11.08 ^bA^	162.00 ± 7.50 ^bA^	391.00 ± 3.01 ^cB^	148.00 ± 2.81 ^bA^

CEM: conventional extraction method; UAE: ultrasound-assisted extraction; MAE: microwave-assisted extraction. DW: distilled water; E-60°: Ethanol 60°; NaDESs LGH (lactic acid: glucose, molar ratio 5:1), CAS (sucrose: citric acid, molar ratio 1:1), CU (choline chloride: urea, molar ratio 1:2) and FGS (fructose: glucose: sucrose, molar ratio 1:1:1). Equal letters indicate no statistically significant difference (uppercase and lowercase letters are for differences between extraction methods and extraction solvents, respectively, according to Tukey’s test (*p* ≤ 0.05)). The analyses were performed for phenolic compounds and flavonoids, independently.

**Table 2:** Content of chalcones in *Z. punctata* extracts obtained with different solvents and conventional and non-conventional extraction methods.

Solvents	Extraction Methods					
	CEM	MAE	UAE	CEM	MAE	UAE
	µg DHC/mL			µg DHMC/mL		
DW	0.10 ± 0.02 ^aA^	0.44 ± 0.05 ^aA^	0.34 ± 0.02 ^aA^	0.10 ± 0.02 ^aA^	0.43 ± 0.01 ^aA^	0.64 ± 0.08 ^aA^
LGH	100.70 ± 5.56 ^dA^	407.90 ± 13.51 ^fB^	446.90 ± 9.87 ^fB^	127.80 ± 3.32 ^dA^	492.10 ± 8.16 ^fB^	475.40 ± 12.76 ^fB^
CAS	21.40 ± 0.54 ^cA^	28.00 ± 0.66 ^cA^	30.00 ± 3.92 ^cA^	22.65 ± 2.87 ^cA^	30.90 ± 4.51 ^cA^	36.00 ± 5.21 ^cA^
CU	101.16 ± 9.00 ^dA^	69.10 ± 7.33 ^dA^	112.89 ± 14.09 ^dA^	108.03 ± 3.42 ^dB^	65.31 ± 3.70 ^dA^	119.48 ± 8.76 ^dB^
FGS	5.14 ± 1.76 ^bA^	6.60 ± 3.00 ^bA^	3.74 ± 2.34 ^bA^	8.27 ± 1.17 ^bA^	8.47 ± 5.23 ^bA^	6.54 ± 2.00 ^bA^
Vegetal oil	259.30 ± 4.98 ^eA^	216.20 ± 7.76 ^eA^	263.00 ± 2.12 ^eA^	246.00 ± 0.8 ^eA^	315.00 ± 0.95 ^eA^	314.00 ± 6.54 ^eA^
E-60°	513.61 ± 20.13 ^fA^	425.90 ± 10.43 ^fA^	844.30 ± 29.00 ^gB^	551.51 ± 5.98 ^fA^	527.80 ± 3.76 ^fA^	883.00 ± 12.25 ^gB^

2′,4′-dihydroxychalcone (DHC); 2′,4′-dihydroxy-3′-methoxychalcone (DHMC); CEM: conventional extraction; UAE: ultrasound assisted extraction; MAE: microwave assisted extraction. DW: distilled water, E-60°: Ethanol 60°, NaDESs: LGH (lactic acid: glucose), CAS (sucrose: citric acid), CU (choline chloride: urea) and FGS (fructose: glucose: sucrose). Equal letters indicate no statistically significant difference (uppercase and lowercase letters are for differences between extraction methods and extraction solvents, respectively, according to Tukey’s test (*p* ≤ 0.05)).

**Table 3:** Antioxidant activity of *Z. punctata* extracts obtained with conventional and non-conventional solvents.

Solvents	Extraction Methods		
	CEM	UAE	MAE
	SC_50_ (µgGAE/mL)		
DW	1.45 ± 0.03 ^bA^	1.80 ± 0.02 ^bA^	1.51 ± 0.02 ^bA^
E-60°	1.50 ± 0.07 ^bA^	1.83 ± 0.07 ^bA^	1.60 ± 0.13 ^bA^
Oil	2.22 ± 1.11 ^cB^	1.64 ± 0.10 ^bA^	2.35 ± 0.15 ^cB^
LGH	1.00 ± 0.06 ^aA^	0.90 ± 0.10 ^aA^	1.20 ± 0.14 ^aA^
CAS	2.37 ± 0.96 ^dB^	2.30 ± 0.01 ^dA^	3.25 ± 1.03 ^dB^
CU	1.43 ± 0.05 ^bB^	1.08 ± 0.16 ^aA^	1.11 ± 0.07 ^aA^
FGS	1.46 ± 0.11 ^bA^	1.42 ± 0.11 ^bA^	1.53 ± 0.02 ^bA^

SC_50_: Concentration of extracts necessary to eliminate 50% of ABTS; GAE: gallic acid equivalent. DW: distilled water; E-60°: Ethanol 60°, NaDESs: LGH (lactic acid: glucose), CAS (sucrose: citric acid), CU (choline chloride: urea) and FGS (fructose: glucose: sucrose). MAE: microwave-assisted extraction; UAE: ultrasound-assisted extraction; CEM: conventional extraction. Values are reported as mean standard deviation of triplicates. Equal letters indicate no statistically significant difference (uppercase and lowercase letters are for differences between extraction methods and extraction solvents, respectively, according to Tukey’s test (*p* ≤ 0.05)).

**Table 4:** Antifungal activity of extracts of *Zuccagnia punctata* obtained by conventional and non-conventional method using different solvents against the fungus *T. mentagrophytes*.

	MIC Values					
Solvents	CEM		MAE		UAE	
	µg GAE/mL	µg DHC/mL	µg GAE/mL	µg DHC/mL	µg GAE/mL	µg DHC/mL
DW	I	I	I	I	I	I
Ethanol	62.5	19.34	31.17	6.05	15	6.33
Oil	7.5	6.23	7.5	6.23	7.5	6.23
LGH	62.5	8.71	31.17	9.95	31.17	10.26
CU	125	8.84	125	8.84	125	8.84
FGS	I	I	I	I	I	I
CAS	I	I	I	I	I	I
	Reference antifungal compounds					
DHC	6.4					
Ketoconazol	8					

GAE: gallic acid equivalent. DHC: 2′,4′-dihydroxychalcone, I: inactive. ND: non determined.

**Table 5:** Natural deep eutectic solvents (NaDESs) used for extraction.

NaDESs Code	Components	Molar Ratio	pH	Viscosity Pa.s
CU	choline chloride: urea	1:2	6	20.95
LGH	lactic acid: glucose	5:1	2	37
CAS	citric acid: sucrose	1:1	3	140
FGS	fructose: glucose: sucrose	1:1:1	6	720

**Table 6:** Extraction methods of compounds from aerial parts of *Z. punctata.*

Extraction Method		PM:S	Solvent	Extraction Time	T°	Power	Amplitude
Conventional extractions	WE	1:20	DW at 100 °C	10 min	-		
	EE	1:20	Ethanol 60°, vegetal oil and NaDESs.	30 min in ultrasonic bath	40 °C	-	-
Non-conventional extractions	UAE	1:20	Ethanol 60°, DW, vegetal oil and NaDESs.	3 cycles 16 s on and 1 min off.	24 °C 55° C	60 W	40%
	MAE	1:20	Ethanol 60°, DW, vegetal oil and NaDESs.	3 cycles 16 s on and 1 min off.	60 °C	60%	-

MAE: microwave-assisted extraction; UAE: ultrasound-assisted extraction; EE: ethanolic extraction; WE: extraction with water; DW: distilled water; NaDESs; T°: temperature; PM: plant material; S: solvent.

**Table 7:** Analytical method validation.

Analyitical Methods	Reference Compounds	Specificity	Linearity	LOD	LOQ	P	AR
Compounds				µg/mL	µg/mL	(n = 10, %RSD)	%
Phenolic Compounds (Folin Cioacalteau method)	Gallic acid	specific	y = 0.0504x (R^2^ = 0.9994)	0.61	1.84	2.24	97.20 ± 1.38
Flavonoids (Woisky and Salatino method)	Quercetin	specific	y = 0.027x (R^2^ = 0.9995)	0.79	2.39	1.45	98.30 ± 1.27
Chalcone (HPLC -DAD quantification)	DHC	specific	y = 5 × 10^6^x (R^2^ = 0.997)	0.021	0.050	0.31	98.02 ± 1.05
	DHMC	specific	y = 1 × 10^7^x (R^2^ = 0.9991)	0.014	0.041	0.41	98.32 ± 1.00

AR: average recovery or accuracy; LOD and LQD: limits of detection and quantification; R^2^: correlation coefficient; RSD: relative standard deviation; P: precision; 2′,4′-dihydroxychalcone (DHC); 2′,4′-dihydroxy-3′-methoxychalcone (DHMC).

## Data Availability

Data are contained within the article and supplementary materials.

## Supplementary Materials

The following supporting information can be downloaded at: https://www.mdpi.com/article/10.3390/plants13182563/s1, Table S1: Yield of phenolic compounds and flavonoids extraction from plant material collected in 2021; Table S2: Yield of phenolic compounds and flavonoids extraction from plant material collected in 2022; Table S3: Yield of phenolic compounds and flavonoids extraction from plant material collected in 2023; Figure S1: Profile of HPLC-DAD.
