# Supplementary material for: Chemical-Functional Analysis of Extracts Obtained from Zuccagnia punctata Powder Using Green Solvents (NaDESs) in Conjunction with Traditional and Non-Traditional Techniques
Source: Plants (Basel). 2024 Sep 12;13(18):2563. doi: 10.3390/plants13182563 (PMC11435240; doi:10.3390/plants13182563)
Supplement: Supplementary file 1 [file plants-13-02563-s001.zip › plants-3158016-supplementary.pdf]

## Supplementary material

**Table S1.** Yield of phenolic compounds and flavonoids extraction from plant material collected in 2021.

| Solvents | CEM | UAE | MAE | CEM | UAE | MAE |
|---|---|---|---|---|---|---|
| | TPC (µg GAE/mL) | | | TF (µg QE/mL) | | |
| **DW** | 1779.21±7.00$^{aB}$ | 15970.00±10.72$^{aB}$ | 1268.00±10.67$^{aA}$ | 80.80±5.12$^{aA}$ | 91.10±1.02$^{aA}$ | 71.18±0.87$^{A}$ |
| **E-60°** | 6282.00±10.00$^{cB}$ | 6492.05±8.98$^{cB}$ | 5175.50±4.09$^{cA}$ | 535.95±12.82$^{dA}$ | 667.30±2.75$^{dA}$ | 630.60±3.09$^{c}$ |
| **Vegetal oil** | 1644.30±11.67$^{aA}$ | 1343.65±3.88$^{aA}$ | 1725.10±1.92$^{aA}$ | 820.00±9.63$^{fA}$ | 832.90±4.66$^{eA}$ | 813.18±2.45$^{fA}$ |
| **LGH** | 6720.12±5.98$^{cdB}$ | 6366.50±5.00$^{cdB}$ | 5393.59±2.45$^{cdA}$ | 291.50±4.90$^{cA}$ | 295.30±6.00$^{bA}$ | 281.00±0.92$^{cA}$ |
| **CAS** | 5073.00±4.98$^{bB}$ | 5594.67±5.66$^{bB}$ | 3611.2±10.32$^{bA}$ | 58.50±4.80$^{aA}$ | 88.80±1.62$^{aA}$ | 68.20±0.27$^{aA}$ |
| **CU** | 7156.00±6.72$^{dB}$ | 8420.00±4.72$^{dC}$ | 6260.00±7.76$^{dA}$ | 655.83±10.08$^{eA}$ | 810.94±2.90$^{eB}$ | 512.64±1.32$^{dA}$ |
| **FGS** | 5083.70±5.00$^{bcA}$ | 6362.80±8.98$^{bcB}$ | 4260.40±8.21$^{bcA}$ | 169.50±5.87$^{bA}$ | 394.00±2.03$^{cB}$ | 150.71±1.94$^{bA}$ |

CEM: conventional extraction method; UAE: ultrasound assisted extraction; MAE: microwave assisted extraction. DW: distilled water, E-60°: Ethanol 60°; NaDESs LGH (lactic acid: glucose), CAS (sucrose: citric acid), CU (choline chloride: urea) and FGS (fructose: glucose: sucrose). Equal letters indicate no statistically significant difference (uppercase and lowercase letters are for differences between extraction methods and extraction solvents respectively, according to Tukey's test ($p \leq 0.05$)). The analyses were performed for phenolic compounds and flavonoids, independently.

**Table S2.** Yield of phenolic compounds and flavonoids extraction from plant material collected in 2022.

| Solvents | CEM | UAE | MAE | CEM | UAE | MAE |
|---|---|---|---|---|---|---|
| | TPC (µg GAE/mL) | | | TF (µg QE/mL) | | |
| **DW** | 1744.79±9.00$^{aB}$ | 1567.40±6.97$^{aB}$ | 1249.00±9.96$^{aA}$ | 50.87±9.84$^{aA}$ | 79.10±2.55$^{aA}$ | 67.40±0.34$^{aA}$ |
| **E-60°** | 6258.00±6.43$^{cB}$ | 6450.20±11.87$^{cB}$ | 5144.92±6.76$^{cA}$ | 567.99±2.00$^{dA}$ | 653.75±1.78$^{dA}$ | 618.30±2.70$^{c}$ |
| **Vegetal oil** | 1606.70±8.77$^{aA}$ | 1318.70±9.73$^{aA}$ | 1708.50±3.73$^{aA}$ | 793.62±3.87$^{fA}$ | 816.70±5.04$^{eA}$ | 805.51±1.54$^{fA}$ |
| **LGH** | 6700.88±9.65$^{cdB}$ | 6335.00±10.31$^{cdB}$ | 5369.00±6.57$^{cdA}$ | 253.90±10.56$^{cA}$ | 283.50±1.36$^{bA}$ | 277.50±2.00$^{A}$ |
| **CAS** | 5029.20±10.65$^{bB}$ | 5567.00±7.23$^{bB}$ | 3587.7±1.98$^{bA}$ | 46.9±2.80$^{aA}$ | 76.77±4.44$^{aA}$ | 66.80±0.11$^{aA}$ |
| **CU** | 7125.00±7.13$^{dB}$ | 8362.20±3.34$^{dC}$ | 6238.00±10.06$^{dA}$ | 618.19±3.23$^{eA}$ | 794,47±6.38$^{eB}$ | 504.14±2.20$^{dA}$ |
| **FGS** | 5057.30±8.09$^{bcA}$ | 6320.00±9.00$^{bcB}$ | 4238.92±7.82$^{bcA}$ | 154.50±1.45$^{bA}$ | 388.28±1.76$^{cB}$ | 145.20±0.85$^{bA}$ |

CEM: conventional extraction method; UAE: ultrasound assisted extraction; MAE: microwave assisted extraction. DW: distilled water, E-60°: Ethanol 60°; NaDESs LGH (lactic acid: glucose), CAS (sucrose: citric acid), CU (choline chloride: urea) and FGS (fructose: glucose: sucrose). Equal letters indicate no statistically significant difference (uppercase and lowercase letters are for differences between extraction methods and extraction solvents respectively, according to Tukey's test ($p \leq 0.05$)). The analyses were performed for phenolic compounds and flavonoids, independently.

**Table S3.** Yield of phenolic compounds and flavonoids extraction from plant material collected in 2023.

| **Solvents** | **CEM** | **UAE** | **MAE** | **CEM** | **UAE** | **MAE** |
|---|---|---|---|---|---|---|
| | TPC (µg GAE/mL) | | | TF (µg QE/mL) | | |
| **-DW** | 1762±7.21$^{aB}$ | 1582±4.16 $^{aB}$ | 1258±2.08 $^{aA}$ | 65.80±1.00 $^{aA}$ | 85.10±1.00 $^{aA}$ | 69.18±2.51$^{aA}$ |
| **E-60°** | 6270±10.56$^{cB}$ | 6470±5.85 $^{cB}$ | 5160±11.37 $^{cA}$ | 551.80±5.29$^{dA}$ | 660.50±6.24$^{dA}$ | 624.00±0.57$^{c}$ |
| **Vegetal oil** | 1624±6.80 $^{aA}$ | 1331±4.93 $^{a A}$ | 1716±5.50 $^{aA}$ | 806.70±3.60$^{fA}$ | 824.70±2.00$^{eA}$ | 809.40±2.88$^{fA}$ |
| **LGH** | 6710±1.52 $^{cdB}$ | 6350±3.11$^{cdB}$ | 5380±5.13 $^{cdA}$ | 271.00±4.35$^{cA}$ | 289.5±3.00$^{bA}$ | 280.00±3.51$^{cA}$ |
| **CAS** | 5050±0.57 $^{bB}$ | 5580±4.00 $^{bB}$ | 3600±10.53 $^{bA}$ | 52.70±1.52$^{aA}$ | 82.80±5.13$^{aA}$ | 67.50±0.57$^{aA}$ |
| **CU** | 7140±9.00$^{dB}$ | 8390±5.68$^{dC}$ | 6250±0.041 $^{dA}$ | 637.00±15.63$^{eA}$ | 802.50±1.53$^{eB}$ | 508.50±9.84$^{dA}$ |
| **FGS** | 5070±5.68$^{bcA}$ | 6340±10.58$^{bcB}$ | 4250±4.04 $^{bcA}$ | 162.00±4.50$^{bA}$ | 391.00±2.31$^{cB}$ | 148.00±2.51$^{bA}$ |

CEM: conventional extraction method; UAE: ultrasound assisted extraction; MAE: microwave assisted extraction. DW: distilled water, E-60°: Ethanol 60°; NaDESs LGH (lactic acid: glucose), CAS (sucrose: citric acid), CU (choline chloride: urea) and FGS (fructose: glucose: sucrose). Equal letters indicate no statistically significant difference (uppercase and lowercase letters are for differences between extraction methods and extraction solvents respectively, according to Tukey's test ($p \leq 0.05$)). The analyses were performed for phenolic compounds and flavonoids, independently.

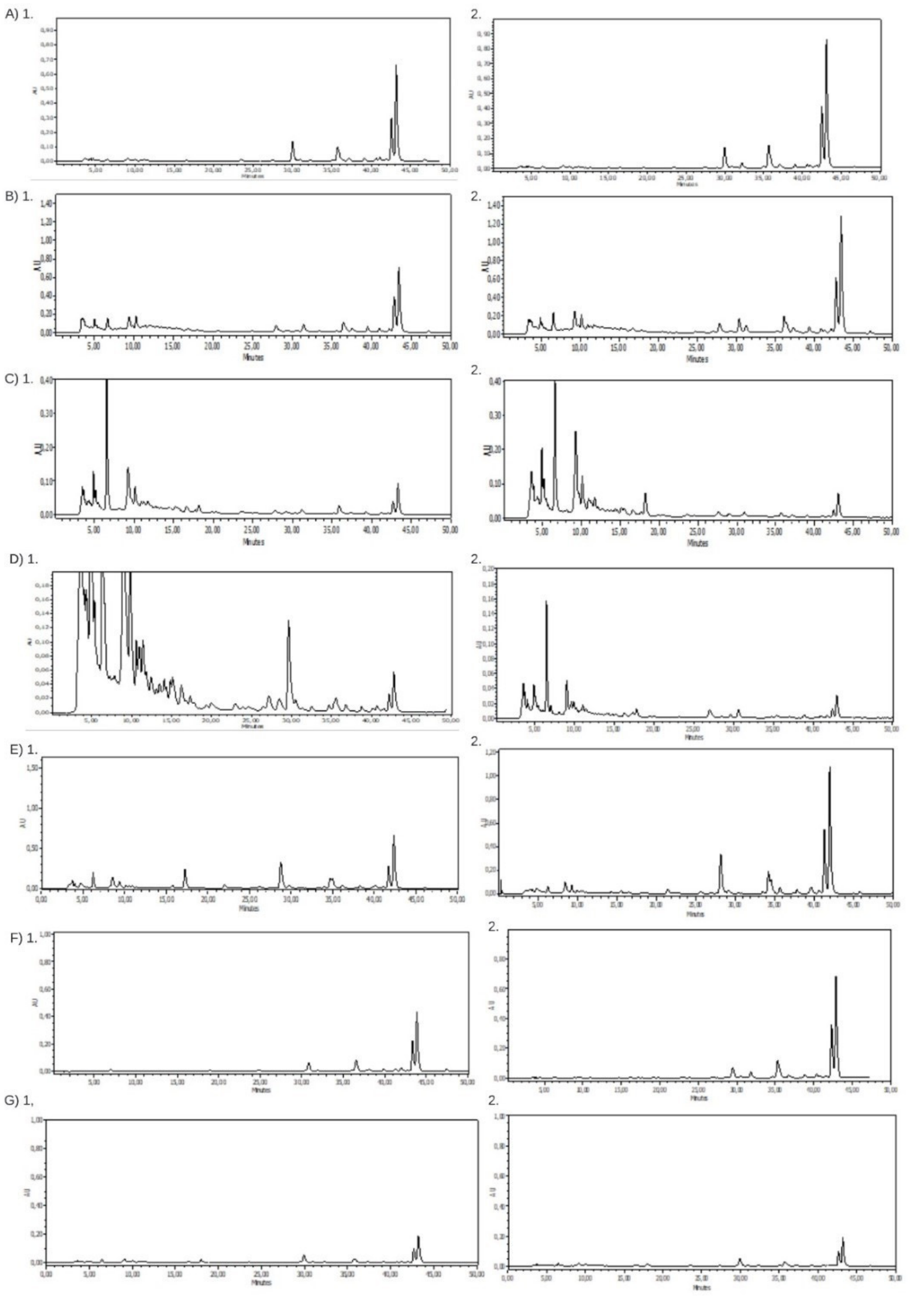


**Figure S1 SM.** Profile of HPLC-DAD. 1) microwave assisted extraction (MAE) and 2) ultrasound assisted extraction (UAE) using different solvents acquired at 330 nm. A) LGH (dilution 1/2), B) CU, C) FGS, D) distilled water, E) Ethanol 60° (dilution 1/2), F) Vegetal oil (dilution 1/7), G) CAS. Peak 1: 2´, 4´-dihydroxy chalcone (DHC); Peak 2: 2´,4´-dihydroxy-3´-methoxychalcone (DHMC).
